# Shear Stress Rescued the Neuronal Impairment Induced by Global Cerebral Ischemia Reperfusion via Activating PECAM-1-eNOS-NO Pathway

**DOI:** 10.3389/fcell.2020.631286

**Published:** 2021-01-21

**Authors:** Jing-Quan Gao, Peng Wang, Jun-Wei Yan, Li-Na Ba, Pi-Long Shi, Hong-Mei Wu, Xue-Ying Guan, Yong-gang Cao, Hong-Li Sun, Xiao-Yuan Mao

**Affiliations:** ^1^Department of Nursing, Harbin Medical University-Daqing, Daqing, China; ^2^Department of Physiology, Harbin Medical University-Daqing, Daqing, China; ^3^Department of Vascular Surgery, The Affiliated Hospital of Qingdao University, Qingdao, China; ^4^Department of Pharmacology, Harbin Medical University-Daqing, Daqing, China; ^5^Institute of Clinical Pharmacology, Xiangya Hospital, Central South University, Changsha, China; ^6^Institute of Clinical Pharmacology, Hunan Key Laboratory of Pharmacogenetics, Central South University, Changsha, China

**Keywords:** global cerebral ischemia reperfusion injury, shear stress, PECAM-1, brain microvascular endothelial cells, nitric oxide

## Abstract

Microvessel hypoperfusion following ischemic stress resulted in a decreased shear stress of brain microvascular endothelial cells (BMECs) and contributed to abnormal expression of PECAM-1 after global cerebral ischemia/reperfusion (I/R) injury. Here, we identified novel pathophysiologic and rehabilitative procedures specific to shear stress in microvascular endothelial cells in response to global cerebral I/R injury. We found that the decrease in cerebral blood flow of gerbils after global cerebral I/R injury reduces shear stress, and the abnormal change in shear stress leads to microvascular endothelial cell and neuron damage. Nevertheless, suitable high levels of shear stress contribute to rescuing the dysfunction and malformation of BMECs via regulating the PECAM-1-eNOS-NO pathway to enhance nitric oxide release, decrease the expression of caspase-3 to reduce apoptosis, and improve the shear-adaptability of endothelial cells, thereby playing a protective role in the gerbil brain.

## Introduction

Ischemic stroke is a prevalent neurological disease with a high rate of death and disability (Naghavi et al., 2015). It has been demonstrated that the microcirculatory dysfunction is a major cause of neuronal impairment, which is closely associated with the subsequent disability and mortality after ischemic stroke (Muoio et al., 2014; Wang et al., 2014). Despite demystifying the pathophysiological mechanisms of cerebral ischemia/reperfusion (I/R) in many publications, the relationship between disturbed cerebral microvessels and neuronal injury is hitherto still not well characterized.

Vascular endothelial cells (VEC) acting as receptors and effectors not only senses the inflammatory signal, shear stress, pressure and other information in the blood, but also responds to this information by secreting a variety of vasoactive substances. For example, when VEC is stimulated following shear stress by blood flow, it can regulate vascular smooth muscle cells by releasing nitric oxide (NO), adjust the vasomotor state of blood vessels, and change blood supply. Blood flow shear stress can be involved in regulating the vascular reconstruction and function by modulating the morphology, differentiation and maturation of VEC, secretion of bioactive substances and cytokines, and vascular permeability (Ives et al., 1986). Physically, the average level of shear stress in the body ranges from 0 to 100 dyn/cm^2^, and the level of shear stress in different parts of the body was disparate.

Various factors are involved in the etiology and pathophysiological effects of I/R injury, such as mitochondrial oxidation and dynamics on endothelial cell function and survival (Wang et al., 2020), edema (Heusch, 2019), microvessel hypoperfusion (Sutherland et al., 2011), reactive oxygen species (ROS) outburst (Sharipov et al., 2014), brain-blood barrier (BBB) disruption (Villringer et al., 2017), and leukocyte infiltration (Uhl et al., 2000). Microvessel hypoperfusion of the ischemic regions has been shown to downregulate shear stress on brain microvascular endothelial cells (BMECs) (Sutherland et al., 2011). Considerable evidence has indicated that endothelial cells are able to directly sense the alterations of stress to regulate vessel endothelial morphology, function, death, and growth and affect disease progression (Na et al., 2008; Chan et al., 2011). Endothelial dysfunction is usually defines as a decreased generation of NO (Scioli et al., 2020). It implicates that deciphering the mechanism underlying the release of NO is extraordinary important for the improvement of endothelial homeostasis, thus alleviating neuronal injury following cerebral I/R insult.

Platelet-endothelial cell adhesion molecule-1 (PECAM-1)/CD31 is a cell adhesion molecule, which is expressed in the endothelial cell intercellular junctions, (Gratzinger et al., 2003; Privratsky and Newman, 2014). Recently, there is evidence supporting that PECAM-1 is also shown to be involved in intracellular signaling which affects the expression and/or activity of endothelial NO synthase, a key enzyme for the modulation of NO release (Park et al., 2015). Additionally, PECAM-1 is also used as an important contributor of the shear sensor and endothelial cell apoptosis (Tzima et al., 2005; Szulcek et al., 2016). Recent studies have shown that shear stress can attenuate rat BMECs (rBMECs) apoptosis under ischemic conditions (Bagi et al., 2005). However, the detailed molecular mechanism remains elusive. Furthermore, studies have indicated that expression of PECAM-1 is abnormal in cerebrovascular endothelial cells after ischemia reperfusion (Gratzinger et al., 2003). However, there is no definite evidence indicating whether the altered expression of PECAM-1 is linked with shear stress during cerebral I/R injury.

Our present work aimed to test the hypothesis that shear stress is a protective factor that restores cerebral I/R-induced neuronal injury by activating the PECAM-1-eNOS-NO pathway in the endothelial cells. We assessed the function of the cerebrovascular system after neuronal injury induced by global cerebral I/R. Mechanistically, we studied the effects of PECAM-1 on the functional changes of gerbils BMECs (gBMECs) by assessing static and variable shear stress values after oxygen-glucose deprivation/reperfusion (OGD/R) injury. To substantiate the in vitro findings, a head-down positioning (HDP) intervention was also used to affect the shear stress, and its effects were assessed in vivo.

## Materials and Methods

### Animals

All animal experiments were approved by the Experimental Animal Ethics Committee of Harbin Medical University and Xiangya Hospital, Central South University, China. The National Institutes of Health Guide for the Care and Use of Laboratory Animals, and the experiments reported are in strict compliance with the ARRIVE guidelines.

Due to the protective effect of estrogen on blood vessels (Nathan and Chaudhuri, 1997; Chambliss and Shaul, 2002), female animals were abandoned and male animals were selected in this study. Adult male Mongolian gerbils (Mongolica gerbillo) (weight, 60–80 g; age, 12–16 weeks) were used for this project. Animals were maintained in a specific pathogen-free laboratory with regular 12/12-h light/dark cycles under controlled temperature and humidity conditions.

### Establishment of Animal Model of Ischemia-Reperfusion

For Mongolian gerbil incomplete cerebral circle of Willis, this was widely used as a global brain I/R model. In this study, adult male Mongolian gerbils (60–80 g) were randomly selected and transient global ischemia was induced as described previously with minor modification (Cao et al., 2011). Gerbils were anesthetized by intraperitoneal injection of 1% (v/w) pentobarbital sodium. After anesthesia, transient global ischemia was induced by five-minnon-invasive microartery clamp occlusion of bilateral common carotidarteries. Restoration of blood flow was obtained post 5-min occlusion. The gerbils were placed on an electric blanket 2 h to prevent hypothermia. Sham-treated animals were treated similarly, except the bilateral common carotidarteries not being occluded.

### Head-Down Positioning Intervention

Gerbils were randomly divided into five groups, and experimental procedures were performed in a blind fashion: sham and days 1, 7, 14, and 21 post ischemia. The gerbils were then sacrificed at days 1, 7, 14, and 21 post ischemia for different experiments.

**FIGURE 1:**
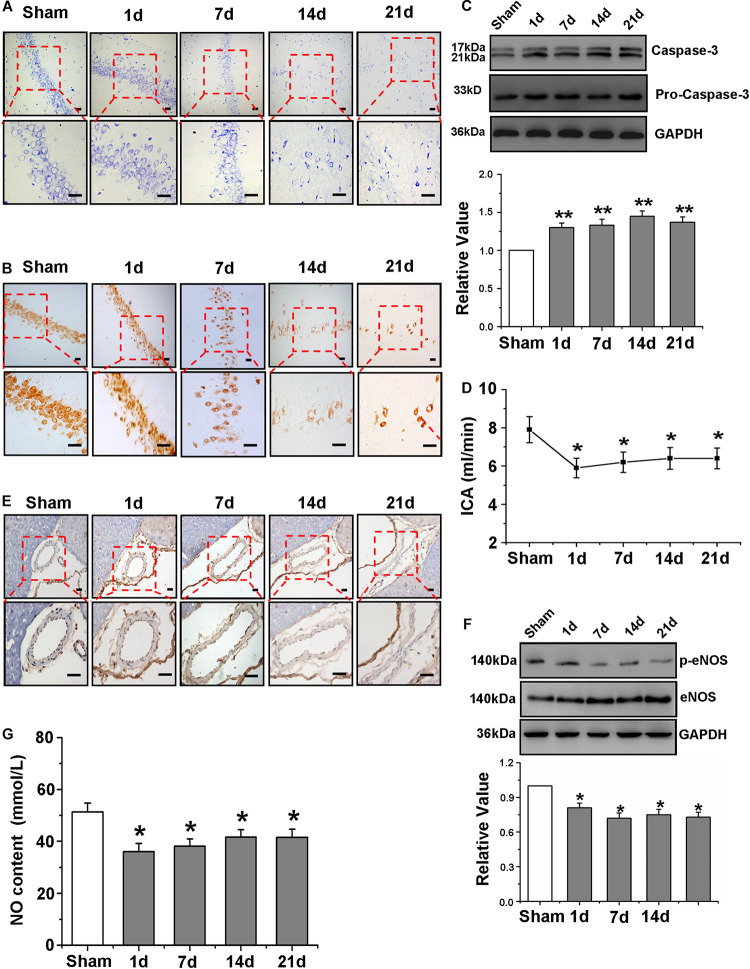
Neurological deficit accompanied by impaired cerebrovascular function after Global Cerebral Ischemia/Reperfusion. **(A)** Representative photomicrographs of gerbils of neuronal loss on brain coronal sections of hippocampal CA1 region by Nissl staining (blue) (×200 and ×400). **(B)** Representative photomicrographs of gerbils of mature neurons on hippocampal CA1 region coronal sections by NeuN immunohistochemistry (brown) (×200 and ×400). **(C)** Detection and quantification of caspase-3 protein expression of hippocampus of gerbils by western blot. **(D)** Detection of the flow velocity of ICA. **(E)** The observation of eNOS positive cells of ICA (dark brown indicates eNOS-positive cells) by immunohistochemistry. **(F)** Detection and quantification of p-eNOS protein expression of hippocampus by western blot (×40 and ×100). **(G)** Measurement of serum NO content. Data were represented by mean ± SEM (*n* = 6). *P < 0.05 and **P < 0.01 vs. sham group.

Surgery gerbils were then randomly divided into the following groups: I/R, I/R+HDP90° 5 min, I/R+HDP90° 10 min, and I/R+HDP90° 20 min. Per the assigned intervention, the gerbils in the sham group and I/R group were maintained in a 0° HDP with fixators for 10 min, and gerbils of each I/R+HDP group were maintained in a 90° HDP with fixators for 5, 10, or 20 min. Surgery gerbils were then randomly divided into I/R, I/R+HDP30°, I/R+HDP60°, and I/R+HDP90° groups. Per the assigned intervention, the gerbils of the sham group and I/R group were maintained in a 0° HDP with fixators for 10 min, and gerbils of each I/R+HDP group were maintained in a 30°, 60°, or 90° HDP with fixators for 10 min. Sham gerbils were randomized into the sham group and Sham+90° (S+90°) group. Surgery gerbils were randomized into I/R group and I/R+90° group. Per the assigned intervention, the sham group and I/R group gerbils were maintained in the 0° head-down position with fixators for 10 min, and gerbils of the S+90° group and I/R+90° group were maintained in the 90° head-down position with fixators for 10 min. For all experiments, intervention was started on the 7th day postoperatively, twice daily, at 9:00 and 15:00, until the 21st day after ischemia. The Supplementary Information of Figure 1 was provided.

### Neurological Symptoms

Neurological deficit scores, according to the stroke index as described by McGraw with minor modification (McGraw, 1977), were used to assess neurological symptoms after global ischemia before the gerbils were sacrificed as follows: (0) no neurological deficit; (1) hunched posture; (2) ptosis; (3) circling behavior; (4) splayed-out hind limb; (5) seizures. The average score was considered as the degree of neurological impairment. The higher the score, the higher the ischemic damage. Neurological function was evaluated by an investigator blinded to the treatment groups.

### Gerbils Brain Microvascular Endothelial Cells Culture and Treatments

GBMECs were prepared from the gerbils between 1 and 3 days and the frontal lobe cortical tissues of animals were used. The gBMECs were cultured in a 25-mm^2^ corning flask in Dulbecco’s modified Eagle medium (DMEM, 10% normal bovine serum albumin and 100 U/ml penicillin/streptomycin) supplemented with 1% endothelial cell growth supplement (ECGS), 10% fetal bovine serum (FBS), 100 μg/ml penicillin/streptomycin and 2 mM L-glutamine solution. The gBMECs were cultured in a 25 mm^2^ corning flask in Dulbecco’s modified Eagle medium (DMEM, 10% normal bovine serum albumin and 100 U/ml penicillin/streptomycin) supplemented with 1% ECGS, 10% FBS, 100 μg/ml penicillin/streptomycin and 2 mM L-glutamine solution. Between 3rd and 5th generation of cells was used in the subsequent experiment.

### Gerbils Brain Microvascular Endothelial Cells of Oxygen-Glucose Deprivation/Reperfusion

Hypoxic condition was balanced with mixed gas (95% N_2_ and 5% CO_2_), and then the normal medium of gBMECs was discarded and quickly replaced with the saturated hypoxia solution. The gBMECs were placed in the hypoxic incubator (95% N_2_ and 5% CO_2_) at 37°C for incubation for 6 h to establish hypoxia (ischemia) model. After that the hypoxia solution was sucked with a straw and replaced with the medium containing sugar and 10% FBS for normal culture for 12 h to establish the reoxygenation (reperfusion) model.

### Cell Transfection

Cells were transfected with small interfering RNA (siRNA) to silence the expression of PECAM-1 protein. The cells were cultured to 30–50% confluence. Then, 2 μg siRNA and 10 μl X-tremeGenesiRNA Transfection Reagent were mixed, and the mixture was diluted in serum-free Opti-MEM-1 medium, incubated at room temperature for 25 min and added directly onto cells. After transfections, cells were cultured for the shear stress interference test.

### Cell Viability

Cell viability was determined using MTT assays. Cells were seeded into 96-well plates and incubated for 48 h at 80% confluence. After the supernatant was removed, 20 μl MTT dye solution (5 mg/ml) and 200 μl serum-free medium were added. Then, the samples were incubated for 4 h at 37°C, and 150 μl dimethyl sulfoxide was added to dissolve the formazan product in each plate on a concentrating table for 15 min. The optical density was measured with a microplate reader.

### Nissl Staining

Nissl staining was conducted on 5 μm thick hippocampal coronal sections. Tissue sections were rinsed with deionized water and then plunged into 0.1% crystal violet for 15 min at 37°C for Nissl staining. Stained cells were observed and captured by a light microscope. Pictures for qualitative analysis of Nissl staining were captured at 200× and 400× magnification.

### Immunofluorescence and Immunohistochemistry

To image the expression of PECAM-1, NeuN and eNOS, the tissue sections were sequentially processed with 0.3% hydrogen peroxide for 10 min, rinsed with PBS for 5 min and washed three times. Next, sections were incubated overnight with anti-PECAM-1 (1:100, Abcam, Cambridge, MA, United States), anti-NeuN (1:200, Chemicon, CA, United States), and anti-eNOS (1:300, Abcam, Cambridge, MA, United States) antibodies at 4°C. After the primary antibody solution was removed, the tissues were washed with PBS three times (5 min each). Then, the tissues were incubated with secondary antibody for 1 h at room temperature and washed three times with PBS (5 min each). As needed, the tissues were counterstained with DAPI (Beyotime Biotechnology, Shanghai, China) for the identification of nuclei. Pictures for qualitative analysis were obtained at 200× and 400× magnification.

### Immunohistochemistry

To analyze the expression of NeuN and eNOS, the tissue sections were sequentially processed with 0.3% hydrogen peroxide for 10 min, rinsed with PBS for 5 min and washed three times. Next, sections were incubated overnight with anti-NeuN (1:200, Chemicon, CA, United States), and anti-eNOS (1:300, Abcam, Cambridge, MA, United States) antibodies at 4°C. After the primary antibody solution was removed, the tissues were washed with PBS three times (5 min each). Then, the tissues were incubated with secondary antibody for 1 h at room temperature and washed three times with PBS (5 min each). Pictures for qualitative analysis were taken at 200× and 400× magnification.

### Immunofluorescence Staining

Immunofluorescence assay was performed by using antibody PECAM-1. Briefly, Brain tissue section and gBMCEs were fixed with 4% paraformaldehyde, permeabilized using 0.2% Triton X-100, incubated with anti-PECAM-1 (1:100, Abcam, Cambridge, MA, United States) at 4°C overnight. Thereafter the samples were washed three times with PBS and incubated with FITC conjugated species-specific secondary antibody. Then, DAPI (Beyotime Biotechnology, Shanghai, China) was counter stained for the identification of nucleus. Moreover, TUNEL staining was conducted according to the manufacturer’s protocol for the *in situ* Cell Death Detection Kit (Roche, Germany), followed by antibody staining against NeuN (1:200, Chemicon, CA, United States). Finally, the sections and cells were observed under a fluorescence microscope. Pictures of 200× and 400× magnification were obtained for quantitative analysis.

### Western Blot Analysis

Tissues extracted from gerbil cell cultures were harvested after 24 h, and the tissue lysates were prepared from samples of hippocampus and cells both homogenizing in ice-cold RIPA lysis buffer. Then, the protein homogenates were separated by sodium dodecyl sulfate polyacrylamide gel electrophoresis and electro blotted onto NC membranes. After the membranes were blocked in 5% nonfat dry milk for 1 h, the membranes were incubated with anti-caspase-3 (1:1000, Cell Signal, United States), anti-GAPDH (1:2000, Santa Cruz, United States), anti-PECAM-1 (1:300, Abcam, Cambridge, MA, United States), anti-eNOS (1:300, Abcam, Cambridge, MA, United States), anti-p-eNOS (1:300, Abcam, Cambridge, MA, United States), or anti-β-actin (1:2000, Santa Cruz, United States) at 4°C overnight, followed by washing and incubation with the secondary antibody for 2 h for adequate conjugation. Finally, immunoreactive bands were visualized using an enhanced chemiluminescence (ECL) kit (Pierce, CA, United States) and exposed on an X-ray film.

### Morris Water Maze Test

The Morris water maze (MWM) test is an established task of spatial learning and reference memory. The MWM apparatus (TMG Technology, Chengdu), a core component of it was a white circular pool with a diameter of 120 cm and a height of 45 cm, placed in an experimental room. The pool was divided into four equal quadrants. Four different visual cues were mounted on the four directions of pool inside. Opaque water was filled in the pool to a height of 25 cm; the water temperature was 21–22°C. A 10 cm diameter platform was located fixedly in the center of a quadrant of the tank and submerged 1.5 cm below the water surface. A video camera was mounted above the center of the tank to record all trials. The gerbils were trained and tested in a MWM as previously described (Gao et al., 2016). On the 1st day, all gerbils were allowed to swim freely for 120 s in order to be familiar with the novel environment of the maze and to locate and climb onto the escape platform. The purpose of this procedure was to decrease the level of stress in rats during MWM task. The experiment was conducted daily between 8 and 10 am and 14 to 16 pm. On the 2nd-4th day, the gerbils were accommodated to find the hidden platform. On the 5th–8th day, each gerbil was subjected to four trials per day in a maximum of 60s. The time to climb onto the platform was recorded as the escape latency (s) for each trial. On the 9th day, the platform was removed, and the passing times and the total swimming path were recorded.

### Shear Stress and Analysis of Shear Adaptation

To simulate the appropriate shear stress of environment in vivo and to better observe the effect of OGD/R on gBMECs, the ibidi system (Integrated BioDiagnostics, Munich, Germany) with a pulse frequency of 60 Hz unidirectional fluid flow was used. Besides this, an air pump controlled by a computer, two medium filled reservoirs and a special four-fold valve set composed the system. Cells were seeded on ibidiu-slides I 0.6 luer for the application of laminar flow, cultured under the conditions of 0, 1, 2, 4, and 6 dyn/cm^2^ incubated for 0, 6, 12, 24, and 48 h, and then cell viability was observed by MTT assay. Based on cell morphology and orientation, shear-adaptation was quantified from phase-contrast images using Photoshop CS6 (Adobe, San Jose, CA, United States). Hereafter, 100 cells from three loci of the monolayers were observed and analyzed under different conditions and time points. Full shear adaptation was defined as a majority of 60% of all cells oriented within an angle of ≤30° and at least 75% of cells elongated (a length-to-width ratio >2). Figure 2 of Supplementary Materials is for the specific experimental process.

**FIGURE 2:**
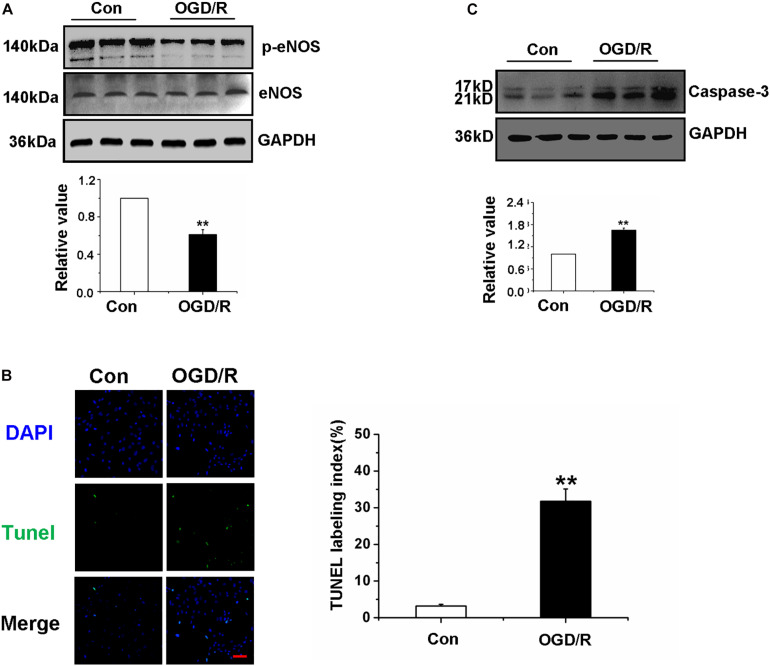
Oxygen-glucose deprivation/reperfusion decreased the function of Gerbils Brain Microvascular Endothelial Cells. **(A)** Detection and quantification of p-eNOS protein expression of gBMECs by western blot. **(B)** Effects of OGD/R on gBMECs apoptosis were detected by TUNEL. Representative images were shown. Cell nuclei were identified using DAPI (blue), and TUNEL-positive cells were indicated by green (×200). **(C)** Detection and quantification of caspase-3 protein expression of gBMECs after OGD/R by western blot. Data were represented by mean ± SEM (*n* = 3). **P < 0.01 vs. control group.

### Blood Flow Velocity Determination

To detect blood flow velocity of the internal carotid artery (ICA), gerbils were prepared for small animal ultrasound imaging. Blood flow through the RCCA was recorded with a high-frequency ultrasound scanner (Vevo 2100, VisualSonics Inc., Toronto, Ontario, Canada).

### Nitrite Detection

Nitric oxide levels in supernatants were detected by testing the levels of nitrite and nitrate with copper-cadmium alloy (Cu–Cd alloy), followed by the detection of the stable degradation products of NO (Griess Reaction NO Assay Kit; Calbiochem, San Diego, CA, United States). The amount of nitrite in cells was determined and normalized to the total protein of cells (pmol/mg protein).

### Statistical Analysis

All data are reported as the mean ± standard error of mean (SEM). The statistical significance of the differences between the groups was evaluated by the independent samples Student’s *t*-test, one-way ANOVA with post hoc Turkey-Kramer test. Differences with a *p*-value below 0.05 were considered statistically significant.

## Results

### Neurological Deficit Accompanied by Impaired Cerebrovascular Character and Function After Global Cerebral Ischemia/Reperfusion

To observe the influence of global cerebral I/R on gerbils over time, the neurological scores were evaluated at days 1, 7, 14, and 21 after ischemia. As illustrated in Table 1, there were no abnormal neurological symptoms in sham gerbils. Global cerebral I/R gerbils at 1, 7, 14, and 21 days all exhibited characteristics of I/R injury with a high neurological deficit score (Table 1).

**TABLE 1:** The neurological deficit scores in gerbils after global cerebral I/R.

Groups	Gerbil number (*n*)	Neurological deficit scores
Sham	10	0.00 ± 0.00
1 day	10	2.33 ± 1.04*
7 days	10	3.50 ± 1.10*
14 days	10	3.33 ± 1.23*
21 days	10	2.67 ± 1.29*

Neurological deficit scores of gerbils were assessed after ischemia surgery at different times according to McGraw’s method. Sham; 1 day, assessed on the 1st day after ischemia surgery; 7 day, assessed on the 7th day after ischemia surgery; 14 day, assessed on the 14th day after ischemia surgery; 21 day, assessed on the 21st day after ischemia surgery. *p < 0.05 vs. sham group.

Ischemia provoked a pronounced neuronal loss in gerbils, especially over time after I/R. As shown in Figures 1A,B, from the 14th day after I/R, there was a downward trend in viable neurons in the hippocampal CA1 region, and significant differences were revealed among the groups. Moreover, quantitative analysis of caspase-3 protein in the gerbil hippocampus revealed that cell apoptosis was increased after global cerebral I/R (Figure 1C).

To investigate the effects of global cerebral I/R on cerebrovascular character and function, the flow velocity of ICA was first detected. The velocity in the sham group did not change significantly during the period of observation; nevertheless, compared with that of the sham group, the blood flow velocity of ICA of the ischemic group was significantly slower beginning at the 1st day after I/R (Figure 1D). In addition, in order to further observe the effect of I/R on vascular morphology, we used eNOS immunohistochemical method to label ICA, and the results of Figure 1E showed the blood vessel of I/R group was not round and the form changed obviously.

Next, p-eNOS protein expression in the hippocampus was determined by western blot to further explore NO production in cerebrovascular endothelial cells, and the results presented in Figure 1F suggest that p-eNOS expression was drastically reduced in the ischemic group beginning at the first day after I/R. Considerable literature has shown that the production of NO by endothelial cells determines changes in the intraluminal diameter of the vessels (Muoio et al., 2014); therefore, the quantification of NO in cerebrovascular endothelial cells is very important to explore the effects of global cerebral I/R on cerebral microvasculature function. The quantization of NO in serum was detected using a kit (Figure 1G), and the results showed that the NO content in serum was decreased significantly beginning at the 1st day after I/R.

### Oxygen-Glucose Deprivation/Reperfusion Decreased the Function of Gerbils Brain Microvascular Endothelial Cells

To investigate whether OGD/R affects the function of gBMECs, gBMECs that underwent OGD/R were employed in in vitro experiments. First, the expression of p-eNOS was detected in the different gBMECs groups to investigate whether OGD/R affects gBMECs function, and the results showed a dramatic decrease in expression in the OGD/R group (Figure 2A). For further exploration of the effect of OGD/R on gBMECs, apoptosis was measured by TUNEL staining, and caspase-3 expression was assessed. TUNEL staining showed that more TUNEL-positive neurons (in green fluorescence) were found in the OGD/R group (Figure 2B), and the expression of caspase-3, as assessed by western blot, was also increased in the OGD/R group (Figure 2C). These observations strongly suggest that OGD/R induced apoptosis of endothelial cells and weakened the function of gBMECs.

### High Shear Stress Played a Protective Role in Gerbils Brain Microvascular Endothelial Cells After Oxygen-Glucose Deprivation/Reperfusion

**FIGURE 3:**
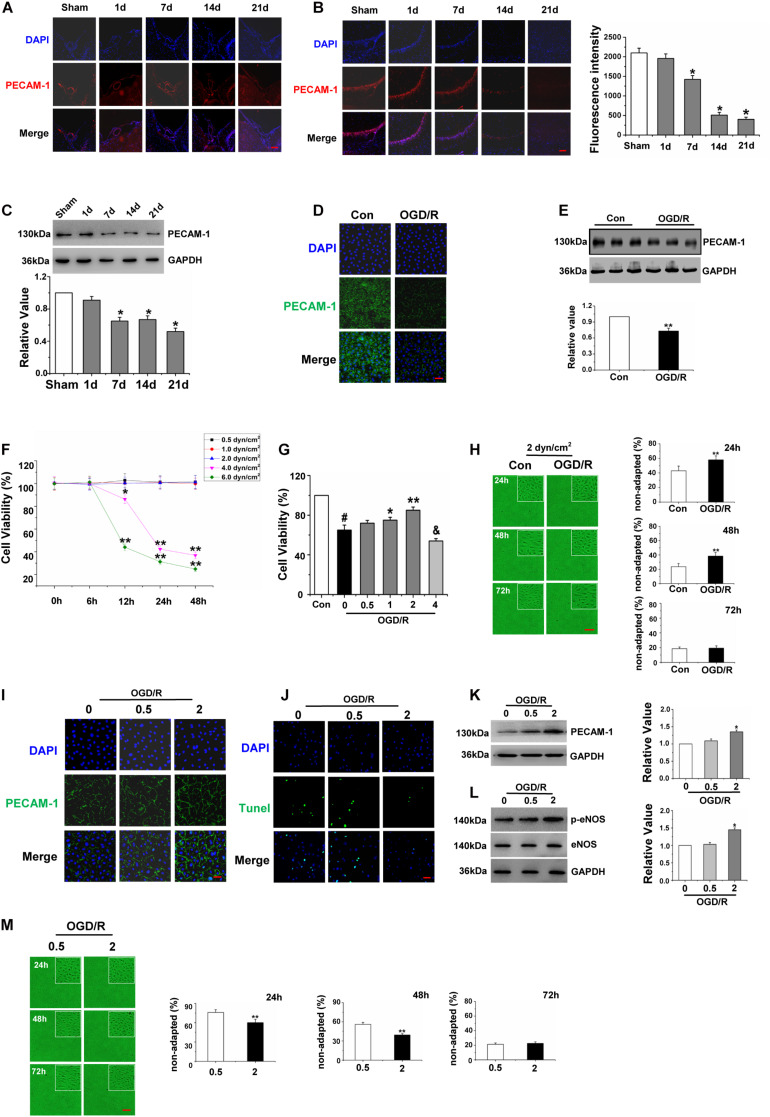
High shear stress played a protective role in Gerbils Brain Micro vascular Endothelial Cells after Oxygen-Glucose Deprivation/Reperfusion. **(A)** The expression levels of PECAM-1 of ICA were assessed to observe the vascular character by immunofluorescence (red), and cell nuclei were identified using DAPI (blue) (×200). **(B)** Distribution and expression levels of PECAM-1 on hippocampal CA1 region were assessed by immunofluorescence (red), and cell nuclei were identified using DAPI (blue) (×200). **(C)** Detection and quantification of PECAM-1 protein expression of hippocampus by western blot, data were represented by mean ± SEM (*n* = 6). *P < 0.05 vs. sham group. **(D)** Effects of OGD/R on the distribution of PECAM-1 of gBMECs (green), and cell nuclei were identified using DAPI (blue) (×200). (E) Detection and quantification of PECAM-1 protein expression of gBMECs by western blot, data were expressed as mean ± SEM (*n* = 3). **P < 0.01 vs. control group. **(F)** Effects of different shear stress on the cell viability of gBMECs at different time points. Data were expressed as mean ± SEM (*n* = 3). *P < 0.05, **P < 0.01 vs. the number of total cells of control gBMECs. **(G)** Effects of different shear stress on the cell viability of gBMECs after cultured 48 h. Data were expressed as mean ± SEM (*n* = 3). ^#^*P* < 0.05 vs. control group. *P < 0.05 and **P < 0.01 vs. OGD/RgBMECs cultured under 0 dyn/cm^2^. **(H)** Effects of OGD/R on the shear-adaption of gBMECsunder 2 dyn/cm^2^. Representative images of shear-adaption of gBMECs after cultured 24, 48, and 72 h under 2 dyn/cm^2^ condition on the left. Statistical analysis of the non-adapted cells after cultured 24, 48, and 72 h on the right. Data were represented by mean ± SEM (*n* = 3) (×100). **P < 0.01 vs. control group. **(I)** Distribution and expression levels of PECAM-1 in gBMECs were assessed by immunofluorescence (green) under 0.5 and 2 dyn/cm^2^. Cell nuclei were identified using DAPI (blue) (×200). **(J)** Effects of OGD/R on gBMECs apoptosis were detected by TUNEL under 0.5 and 2 dyn/cm^2^ condition. Representative images were shown. Cell nuclei were identified using DAPI (blue). TUNEL-positive cells were indicated by green (×200). **(K)** Detection and Quantification of PECAM-1 protein expression of OGD/R gBMECculturedunder 0.5 and 2 dyn/cm^2^ condition by western blot. Data were represented by mean ± SEM (*n* = 3). *P < 0.05 vs. OGD/R gBMEC cultured under 0 dyn/cm^2^condition. **(L)** Detection and quantification of p-eNOS protein expression of OGD/R gBMECcultured under 0.5 and 2 dyn/cm^2^ condition by western blot. Data were represented by mean ± SEM (*n* = 3). *P < 0.05 vs. OGD/R gBMEC cultured under 0 dyn/cm^2^condition. **(M)** Effects of low (0.5 dyn/cm^2^) and high (2 dyn/cm^2^) shear stress on the shear-adaption of OGD/R gBMEC. Representative images of shear-adaption of OGD/R gBMEC on the left. Statistical analysis for the non-adapted cells at 24, 48, and 72 h on the right. Data were represented by mean ± SEM (*n* = 3). **P < 0.01 vs. OGD/R gBMEC cultured under 0.5 dyn/cm^2^condition (×100).

We observed PECAM-1 immunofluorescence to further investigate the vascular character. Circle vessels were found in the sham group at baseline. However, after the acute phase of cerebral ischemia followed by reperfusion, cerebrovascular malformation was obviously present beginning at the 7th day after I/R (Figure 3A). Next, to observe the effects of global cerebral I/R on cerebral microvasculature, the microvascular density of the hippocampus CA1 region was detected by detecting changes in PECAM-1 expression. PECAM-1 immunofluorescence revealed a decrease in the number of blood vessels in I/R group compared with that in the sham group, particularly beginning at the 14th after I/R (Figure 3B). Furthermore, the expression of PECAM-1 in the hippocampus was detected by western blot, and the results showed that PECAM-1 expression declined significantly beginning at the 7th day after I/R (Figure 3C). And then, we detected the effect of OGD/R on the quantity of gBMECs, which were identified by the expression of PECAM-1. The immunofluorescence and western blot of PECAM-1 revealed that PECAM-1 expression decreased after OGD/R (Figures 3D,E).

As BMECs inhabit a fluidly dynamic environment in vivo, to better observe the effect of OGD/R on gBMECs, we simulated conditions of fluid dynamics in vitro. To choose suitable shear stress, we cultured normal and OGD/R gBMECs under different conditions of shear stress and then used an MTT assay to detect the cell viability of two types of gBMECs for varying culture durations. As shown in Figure 3F, the MTT assay revealed significant differences in cell viability between the low shear stress (0.5, 1, and 2 dyn/cm^2^) and high shear stress (4 and 6 dyn/cm^2^) conditions after OGD/R at the time points of 0, 6, 12, 24, and 48 h. The shear stress of 0.5, 1, and 2 dyn/cm^2^ did not affect the cell viability of normal gBMECs; nevertheless, if the shear stress was too high (4 and 6 dyn/cm^2^), cell viability decreased at 12, 24, and 48 h. Furthermore, after culture for 48 h, the cell viabilities of OGD/R gBMECs under different shear stress values of 0.5, 1, and 2 dyn/cm^2^ were higher than those under static conditions. However, high shear stress (4 dyn/cm^2^) decreased the cell viability of OGD/R gBMECs (Figure 3G).

For this reason, 2 dyn/cm^2^ was chosen to explore the effects of OGD/R on gBMECs shear adaptation. EC shear adaption is an important factor that affects the function of ECs. We observed the shear responsiveness after OGD/R (Figure 3H) under 2 dyn/cm^2^ of shear stress. The control group responded quickly, and the OGD/R group responded slowly to shear stress. Control cells responded to shear stress with 57.0 ± 6.5% elongated after 24 h, but the response of OGD/R group cells was diminished, as only 42.0 ± 5.5% of the cells elongated after the first 24 h. After 48 h, control cells had reached shear adaptation with 76.4 ± 4.5% of all cells elongated. In contrast, only 62.6 ± 4.8% of all cells had elongated in the OGD/R group. There were no differences in shear adaptation between the two groups until the 72 h time point. Taken together, these data suggested that OGD/R-treated cells exhibit a delay in shear adaptation.

Here, PECAM-1 expression was detected to explore the effect of low and high shear stress that does not affect cell viability on gBMECs. Immunofluorescence staining and western blot of PECAM-1 demonstrated that the expression of PECAM-1 in OGD/R gBMECs was higher under 2 dyn/cm^2^ than under static conditions and 0.5 dyn/cm^2^ (Figures 3I,K). Furthermore, TUNEL staining revealed that the apoptosis of OGD/R gBMECs was significantly lower under 2 dyn/cm^2^ than under the other conditions tested (Figure 3J). Moreover, by western blot, we found that the expression of p-eNOS was increased under 2 dyn/cm^2^ compared to that under the other conditions (Figure 3L). Furthermore, the shear responsiveness of OGD/R gBMECs under different levels of shear stress (0.5 and 2 dyn/cm^2^) was observed, and the results showed that the shear adaption had advancing shear responsiveness at 2 dyn/cm^2^ (Figure 3M) at 24, 48, and 72 h. Therefore, we inferred that appropriately high levels of shear stress, without sacrificing cell viability, provide better protection for OGD/R gBMECs.

### High Shear Stress Restored Oxygen-Glucose Deprivation/Reperfusion Gerbils Brain Microvascular Endothelial Cells Function Through the PECAM-1-eNOS Signal Pathway

**FIGURE 4:**
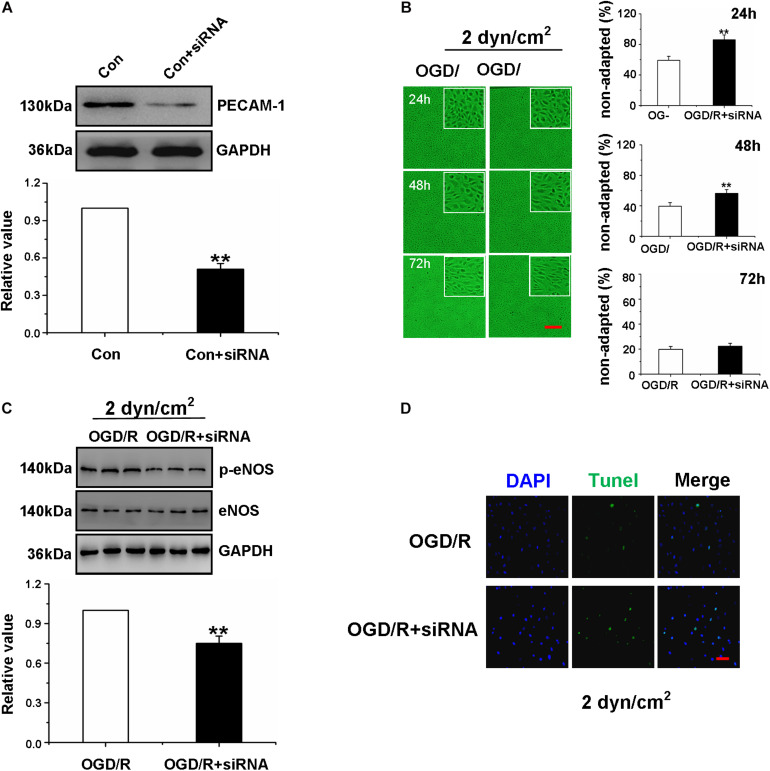
High shear stress restored Oxygen-Glucose Deprivation/Reperfusion Gerbils Brain Microvascular Endothelial Cells function through the PECAM-1-eNOS signaling pathway. **(A)** Western blotting analysis of PECAM-1 protein levels of gBMEC in control group and siPECAM-1 group. **P < 0.01 vs. control group. **(B)** The shear-adaptation of gBMEC with siRNA PECAM-1 after OGD/R. Representative images of shear-adaption of gBMEC on the left. Statistical analysis for the non-adapted cells at 24, 48, and 72 h on the right. The data were expressed as mean ± SEM (*n* = 3) (×100). **P < 0.01 vs. OGD/R group. **(C)** Western blotting analysis of eNOS protein levels of gBMECs cultured under 2 dyn/cm^2^ condition in OGD/R group and OGD/R+ siPECAM-1 group. The data were expressed as mean ± SEM (*n* = 3). **P < 0.01 vs. OGD/R group. **(D)** Effects of siPECAM-1 on OGD/R gBMECsapoptosis were detected by TUNEL cultured under 2 dyn/cm^2^ condition. Representative images were shown. Cell nuclei were identified using DAPI (blue). TUNEL-positive cells were indicated by green (×200).

To determine if PECAM-1 mediated shear adaptation of gBMECs and contributed to the above observed responses, PECAM-1 expression was silenced (using siRNA) in normal gBMECs. Western blot analysis indicated that siRNA transfection significantly reduced EC PECAM-1 expression and that PECAM-1 silencing using siRNA was effective (Figure 4A). Following successful siRNA transfection in normal gBMECs, PECAM-1 silencing was performed in OGD/R gBMECs. First, we observed the shear responsiveness of gBMECs cultured under 2 dyn/cm^2^, and after PECAM-1 expression was silenced, OGD/R gBMECsno longer responded to the high shear stress that had been previously shown to advance the shear responsiveness of OGD/R gBMECs (Figure 4B).

To further explore the effect of siRNA transfection in OGD/R gBMECs, western blot analysis of p-eNOS expression and TUNEL staining were measured. The results showed that the expression of p-eNOS was decreased and the number of TUNEL-positive gBMECs was increased in OGD/R gBMECs with PECAM-1 siRNA compared with those in OGD/R gBMECs (Figures 4C,D). Taken together, the results in Figures 4A–D demonstrated that when PECAM-1 was absent, the OGD/R-treated gBMECs did not respond to high shear stress; therefore, high shear stress was no longer able to reduce OGD/R-induced gBMECs injury or enhance OGD/R gBMECs function.

### Head-Down Positioning Intervention Increased Shear Stress to Regulate the Cerebrovascular Function of Gerbils After Ischemia/Reperfusion

**FIGURE 5:**
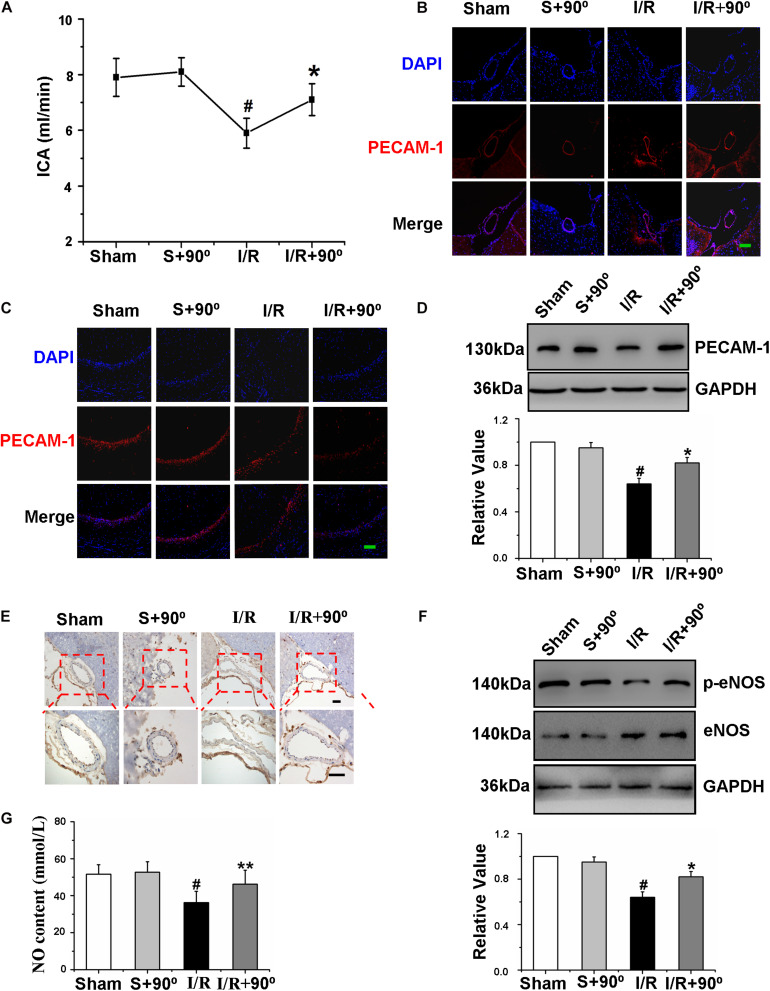
Head-down Positioning increased shear stress to regulate the cerebrovascular function of gerbils after Ischemia/Reperfusion. **(A)** The effects of HDP90° on the flow velocity of ICA of gerbils after I/R. **(B)** The effects of HDP90°on the expression levels of PECAM-1 of ICA were assessed to observe the vascular character of it by immunofluorescence (red) (×200). Cell nuclei were identified using DAPI (blue). **(C)** The effects of HDP90°on the expression levels of PECAM-1 of hippocampal CA1 region were assessed by immunofluorescence (red) (×200). Cell nuclei were identified using DAPI (blue). **(D)** The effects of HDP90° on the expression of PECAM-1 of hippocampus of gerbils after I/R by western blotting. **(E)** The effects of HDP90° on the expression of eNOS (dark blue indicates eNOS-positive cells) of gerbils after I/R by Immunohistochemistry (×40 and ×100). **(F)** The effects of HDP90° on the expression of p-eNOS of gerbils after I/R by western blotting. **(G)** The effects of HDP90° on serum’s NO content of gerbils after I/R. Data were represented by means ± SEM (*n* = 6). ^#^*P* < 0.05 vs. sham group.*P < 0.05 and **P < 0.01 vs. I/R group.

Exercise changes the hemodynamics of the body (Connes et al., 2012), and whether the HDP intervention influenced shear stress to regulate cerebrovascular functions in I/R gerbils was uncertain. We detected the ICA blood flow velocity in vivo to investigate whether the HDP intervention affected the shear stress of I/R gerbils, and the result confirmed that the HDP intervention increased the ICA blood flow velocity (Figure 5A). The ICA is the major blood vessel that supplies cerebral blood flow, and its contractile state and shape seriously affect cerebral blood flow volume. To further observe the effects of the HDP intervention on ICA character, immunofluorescence of PECAM-1 and immunohistochemistry of eNOS were used. The results of Figures 5B,E showed that the cerebrovascular malformation induced by I/R was reversed after the HDP intervention. Additionally, exploring the effects of the HDP intervention on cerebrovascular endothelial cell function was crucial. Firstly, to determine the effect of the HDP intervention on cerebrovascular density in I/R gerbils, we identified microvessels of the hippocampus by the expression of PECAM-1, and immunofluorescence staining and western blot of expression of PECAM-1 revealed that the cerebrovascular density of the hippocampus in I/R gerbils significantly increased after 3 weeks of the HDP intervention (Figures 5C,D). Second, we further detected the expression of p-eNOS and NO content to observe the effect of HDP on the vascular function of gerbils after I/R. As illustrated in Figures 5F,G, the HDP intervention rescued the expression of p-eNOS (Figure 5F). The results showed that HDP treatment increased the NO content in the serum of I/R gerbils (Figure 5G).

### Head-Down Positioning Intervention Rescued the Learning and Memory Ability of Gerbils After Ischemia/Reperfusion

**FIGURE 6:**
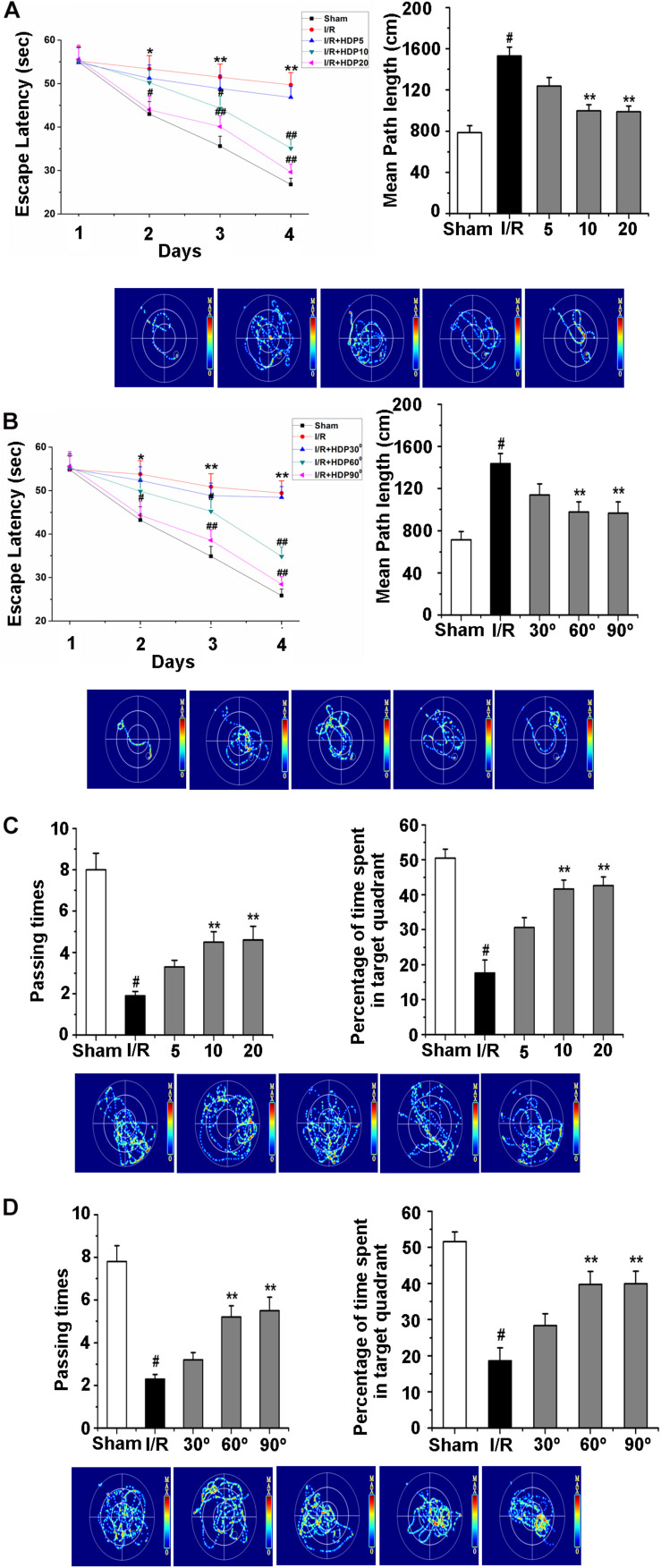
Head-down Positioning reversed the learning and memory deficits in gerbils after Ischemia/Reperfusion. **(A)** The escape latency time (upper left) and escape latency distance (upper right) of gerbils after I/R for HDP90° for 5, 10, and 20 min by morris water maze test. **(B)** The escape latency time (upper left) and escape latency distance (upper right) of gerbils after I/R of 30°, 60°, and 90° for HDP for 10 min by morris water maze test. **(C)** The number of passing times (upper left) and percentage of time spent in target quadrant (upper right) of gerbils after I/R for HDP90° for 5, 10, and 20 min by morris water maze test. **(D)** The number of passing times (upper left) and percentage of time spent in target quadrant (upper right) of gerbils after I/R of 30°, 60°, and 90° of HDP for 10 min by morris water maze test. Data were represented by mean ± SEM (*n* = 6). ^#^*P* < 0.05 vs. sham group. **P < 0.01 vs. I/R group.

Mounting evidence has suggested that regular exercise can keep the mind sharp and increase learning and memory capacity (Diederich et al., 2017; Lloyd et al., 2017). HDP is a type of exercise that requires no specific location or equipment. Therefore, we examined whether HDP is able to enhance the learning and memory ability of gerbils after I/R. To investigate the effect of HDP, following 2 weeks of HDP training, the MWM was used to test cognitive deficits. First, we analyzed the effects of HDP90° for 5, 10, and 20 min on procedural learning. After I/R, the gerbils showed a decrease in the escape latency and number of platform crosses in the consecutive trials (Figures 6A,C). The HDP90° groups performed significantly better than I/R group, especially the HDP90° groups exposed to HDP90° for 10 min and 20 min (Figures 6A,C). Therefore, 10 min HDP was chosen for the following experiments.

Next, the effect of different angles of HDP on the learning and memory deficits of gerbils after I/R was explored to select the proper angle of HDP. Here, the MWM test was also used to detect the learning and memory deficits of the different groups of gerbils. Statistical analysis of the escape latency and number of pasting times confirmed that the I/R-associated memory deficits were significantly rescued after HDP treatment (30°, 60°, and 90°) for 10 min (Figures 6B,D). These results suggested that HDP may rescue the learning and memory abilities of gerbils after I/R, and HDP 90° for 10 min was the most effective.

### Head-Down Positioning Intervention Played a Protective Role in Hippocampal Neurons of Ischemia/Reperfusion Gerbils

**FIGURE 7:**
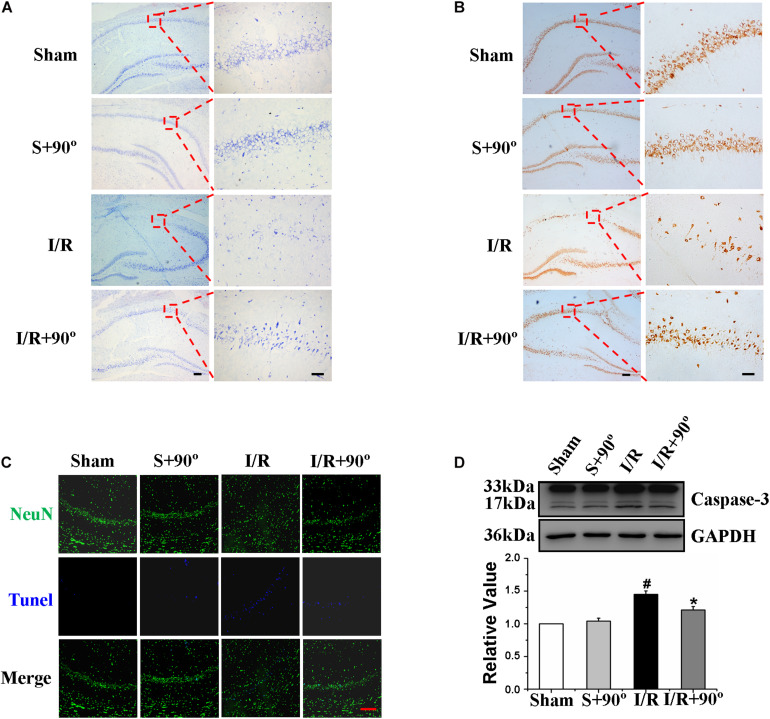
Head-down Positioning played a protective role on hippocampal neurons of Ischemia/Reperfusion gerbils. **(A)** The effects of HDP90° on gerbils’ number of neurons in hippocampal CA1 region after I/R by Nissl staining (×40 and ×200). **(B)** The effects of HDP90° on gerbils’ mature neurons in hippocampal CA1 region after I/R by NeuN immunohistochemistry (×40 and ×200). **(C)** The effects of HDP90° on gerbils’ neuronal apoptosis in hippocampal CA1 region after I/R by NeuN immunoluorescence staining and TUNEL staining (×200). **(D)** The effects of HDP90° on gerbils’ expression of caspase-3 of hippocampus after I/R by western blotting. Data were represented by mean ± SEM (*n* = 6). ^#^*P* < 0.05 vs. sham group. *P < 0.05 and **P < 0.01 vs. I/R group.

The hippocampus is an important region for learning and memory in the brain (Freund and Buzsáki, 1996; Thome et al., 2017); therefore, we investigated the effect of HDP on hippocampal neurons of gerbils after I/R. Nissl staining and NeuN immunohistochemistry were chosen to observe the hippocampal neurons in the different gerbil groups (sham, sham+HDP90°, I/R, I/R+HDP90°). We found that the proportion of neurons that survived was substantially higher and the number of positive cells (dark brown indicates NeuN positive cells) was greater in I/R+HDP90° group than I/R group (Figures 7A,B). Next, we wanted to determine whether HDP plays a protective role in hippocampal neurons of gerbils exposed to I/R injury through an anti-apoptotic mechanism. TUNEL staining and caspase-3 western blot revealed that HDP prevented apoptosis (Figures 7C,D). Therefore, the results shown in Figure 7 indicate that HDP has a positive effect on the damage caused by I/R and protects hippocampal neurons in I/R gerbils.

## Discussion

The present study identified novel pathophysiologic and rehabilitative procedures specific to shear stress in microvascular endothelial cells in response to global cerebral I/R injury. The results showed that the decrease in cerebral blood flow of gerbils after global cerebral I/R injury could reduce shear stress, and the abnormal change in shear stress might lead to microvascular endothelial cell and neuron damage. Nevertheless, suitable high-level shear stress might contribute to rescue the dysfunction and malformation of BMECs via increasing the expression of PECAM-1. Increased PECAM-1 could increase the expression of p-eNOS protein in endothelial cells, which could lead to the enhanced NO release. Additionally, increased PECAM-1 could decrease caspase-3 expression, reduce apoptosis and improve shear adaptation of endothelial cells, which was suggested to play a protective role in the gerbil brain.

Earlier reports have implied that cerebral ischemia reperfusion injury causes a drop in cerebral blood flow and results in persistent damage and dysfunction of cerebral microvascular endothelial cells, accounting for previous reports (Kahl et al., 2018) and our results, including the decrease in the expression of p-eNOS protein (Chien et al., 2015) and NO production. NO is a vasodilator that may lead to increased cerebral perfusion. Meanwhile, cerebral microvascular endothelial cells are an important part of the blood-brain barrier (BBB), and a decline in endothelial cell function also affects the BBB function and material exchange, further aggravating neuronal damage. Therefore, this study focused on cerebral microvascular endothelial cells.

The dysfunction of brain vascular endothelial cells is a critical element in the pathogenesis of global cerebral I/R injury, characterized by loss of vasodilator responses due to a progressive imbalance in favor of endogenous vasodilators and vasoconstrictors such as NO (Liew et al., 2015) and endothelin-1 (ET-1) (Rumaks et al., 2012), which in turn affect the function of various other vascular cells, including smooth muscle cells and pericytes. In addition to dysfunctional vasodilators, vasoconstrictors and growth factor secretion, blood-brain-barrier (BBB) disruption is long believed to be a feature of endothelial dysfunction in I/R injury (Gasche et al., 2001). PECAM-1, as an endothelial junction molecule that contributes to vascular barrier integrity via homotypic binding, has been studied extensively (Hashimoto et al., 2011) and is expressed in endothelial cells, circulating platelets and most circulating leucocytes. Previous findings have suggested that PECAM-1 is also a direct transducer of mechanical forces (Tzima et al., 2005). Our findings are in line with these reports showing that PECAM-1, as a mechanical sensor, can regulate the apoptosis and function of endothelial cells in cerebral ischemia reperfusion injury. In accordance with our findings, research shows that cytosolic PECAM-1 cleavage is caspase mediated (Ilan et al., 2001), and our findings suggest that the link between PECAM-1 and caspase-3 also applies to cerebral ischemia reperfusion injury.

An abnormal EC phenotype indicative of disturbed endothelial shear responses was identified in patients with congenital heart disease and pulmonary arterial hypertension (Szulcek et al., 2016) with severe vascular remodeling (Rabinovitch et al., 1986), but the exact role of this maladaptation of BMECs exposed to transient ischemia and long-term reperfusion injury has remained unknown. Endothelial shear adaptations reflect endothelial function and can be regulated by PECAM-1 (Szulcek et al., 2016). While altered PECAM-1 signaling in global cerebral I/R injury remains to be fully defined, our data strongly implicate a central role for PECAM-1 in the defective OGD/R shear response of gBMECs, as PECAM-1 silencing fully resembled the OGD/R shear phenotype of gBMECs. This observation is consistent with previous findings suggesting that PECAM-1 is a direct transducer of mechanical forces (Tzima et al., 2005) that couples fast temporal shear changes into gBMECs and thereby mediates the timing of NO-dependent vasodilation (Bagi et al., 2005). Nevertheless, to our knowledge, this report represents the first evidence directly linking PECAM-1 to the defective shear responsiveness of gBMECs under conditions of OGD/R.

**FIGURE 8:**
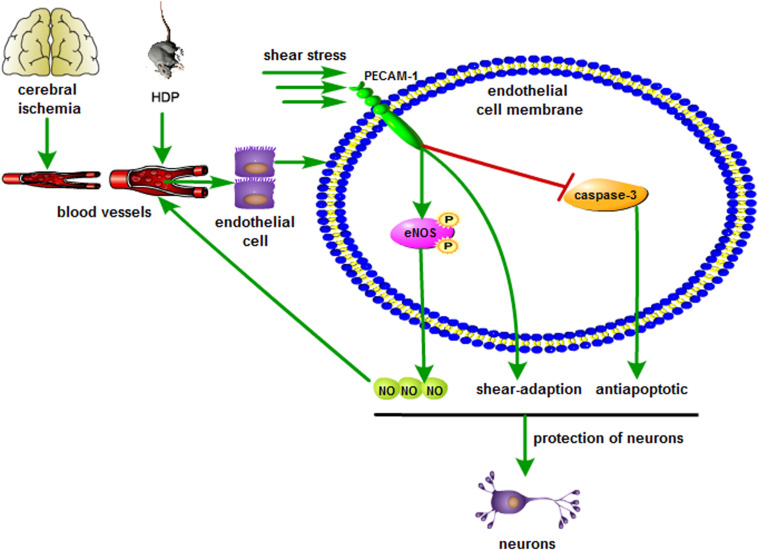
Schematic illustration showing the neuroprotection of shear stress against cerebral ischemia. Appropriate shear stress activates PECAM-1 expression in microvascular endothelial cell, resulting in the up-regulation of p-eNOS and NO release, which further facilitates the blood supply. In addition, shear stress also decreases caspase-3 activity and neuronal apoptosis.

Several publications have described that shear stress generated by cerebral blood flow attenuates rBMECs apoptosis under ischemic conditions (Tian et al., 2013), and shear stress of rBMECs in ischemic regions could be upregulated by increasing CBF (cerebral blood flow) (Sutherland et al., 2011). However, methods to improve the situation have not been identified. We demonstrated that increasing the shear stress of cerebral blood flow to appropriate levels may alleviate the dysfunction and necrosis of microvascular endothelial cells to some extent, as indicated by an increase in PECAM-1 expression, which results in an increase in p-eNOS expression in microvascular endothelial cells and enhances NO release. Increased NO will further dilate adjacent blood vessels in smooth muscles, improve cerebral blood supply, and increase shear stress. We assume that there may be a positive regulatory mechanism between suitable high-level shear stress and NO; furthermore, high-level shear stress has a continuous protective effect on cerebral vessels and neurons of gerbils with global cerebral I/R injury (Figure 8).

BMECs are able to directly sense changes in stress to regulate vessel function and affect disease progression (Na et al., 2008; Chan et al., 2011). First, we used OGD/R-treated gBMECs to detect the influence of proper high-level shear stress on BMECs and to explore whether these factors play a role in BMECs. Here, we demonstrate that properly high-level shear stress could enhance the shear adaptation of OGD/R gBMECs via increasing the expression of PECAM-1, which could reduce apoptosis and increase the expression of p-eNOS in OGD/R gBMECs.

Furthermore, we validated the effect of proper high-level shear stress on transient ischemia and long-term reperfusion injury in vivo. Here, we demonstrate that cognitive impairment in I/R gerbils is associated with a significant decline in microvascular density and p-eNOS expression in the hippocampus, connected with malformation and dysfunction of the major blood vessels that supply cerebral blood flow (Figure 1). Animal studies have suggested that endothelium-dependent vasodilation and CBF could be improved by physical training (Endres et al., 2003; Gertz et al., 2006), and these advantages have also been demonstrated in clinical stroke survivors (Ivey et al., 2011). However, whether exercise rehabilitation plays a protective role in I/R patients by improving the shear stress of CBF remains to be investigated. Shear stress refers to the friction between blood flow and the vascular endothelium, which is closely related to blood characteristics, blood flow velocity and vascular morphology. In micro capillaries, shear stress is mainly related to blood perfusion (McHedlishvili, 1998; Cho and Cho, 2011). HDP90° is a yoga method and is the simplest way to improve CBF. Here, we demonstrate that an HDP90° intervention accelerated the blood flow velocity and improved the vascular morphology of the ICA in I/R gerbils. According to our shear stress calculations, the HDP90° intervention can be inferred to increase the shear stress of cerebral blood flow in I/R gerbils. Furthermore, we found that the protective effects of high-level cerebral blood flow shear stress in I/R gerbils rescued the cognitive impairment of I/R gerbils through anti-apoptotic and anti-oxidative mechanisms. Importantly, the dysfunction of the ICA and micro vascular density and the expression of p-eNOS were alleviated by HDP90°.

In summary, the findings of this study showed that appropriate high-level cerebral blood flow shear stress alleviates cerebral I/R-induced brain injury by improving the function of cerebral microvascular endothelial cells and suggest that shear stress could be a neuroprotective candidate. Our mechanistic insights into the pathological changes associated with global cerebral I/R injury in gerbils and shear stress of cerebral blood flow. This study could supply the basis for the development of a novel potential clinical rehabilitation strategy of patients with I/R injury and provide an idea for the establishment of new possible intervention methods.

## Data Availability Statement

The raw data supporting the conclusions of this article will be made available by the authors, without undue reservation.

## Ethics Statement

The animal study was reviewed and approved by Experimental Animal Ethics Committee of Harbin Medical University and Xiangya Hospital, Central South University, China.

## Author Contributions

YC, H-LS, and X-YM designed the manuscript. J-QG, PW, and J-WY carried out the experiments and wrote the manuscript. L-NB, P-LS, H-MW, and X-YG analyzed the data. X-YM revised the manuscript. All authors contributed to the article and approved the submitted version.

## Conflict of Interest

The authors declare that the research was conducted in the absence of any commercial or financial relationships that could be construed as a potential conflict of interest.
