# Supplementary figures and images for: Shear Stress Rescued the Neuronal Impairment Induced by Global Cerebral Ischemia Reperfusion via Activating PECAM-1-eNOS-NO Pathway
Source: Front Cell Dev Biol. 2021 Jan 21;8:631286. doi: 10.3389/fcell.2020.631286 (PMC7859356; doi:10.3389/fcell.2020.631286)

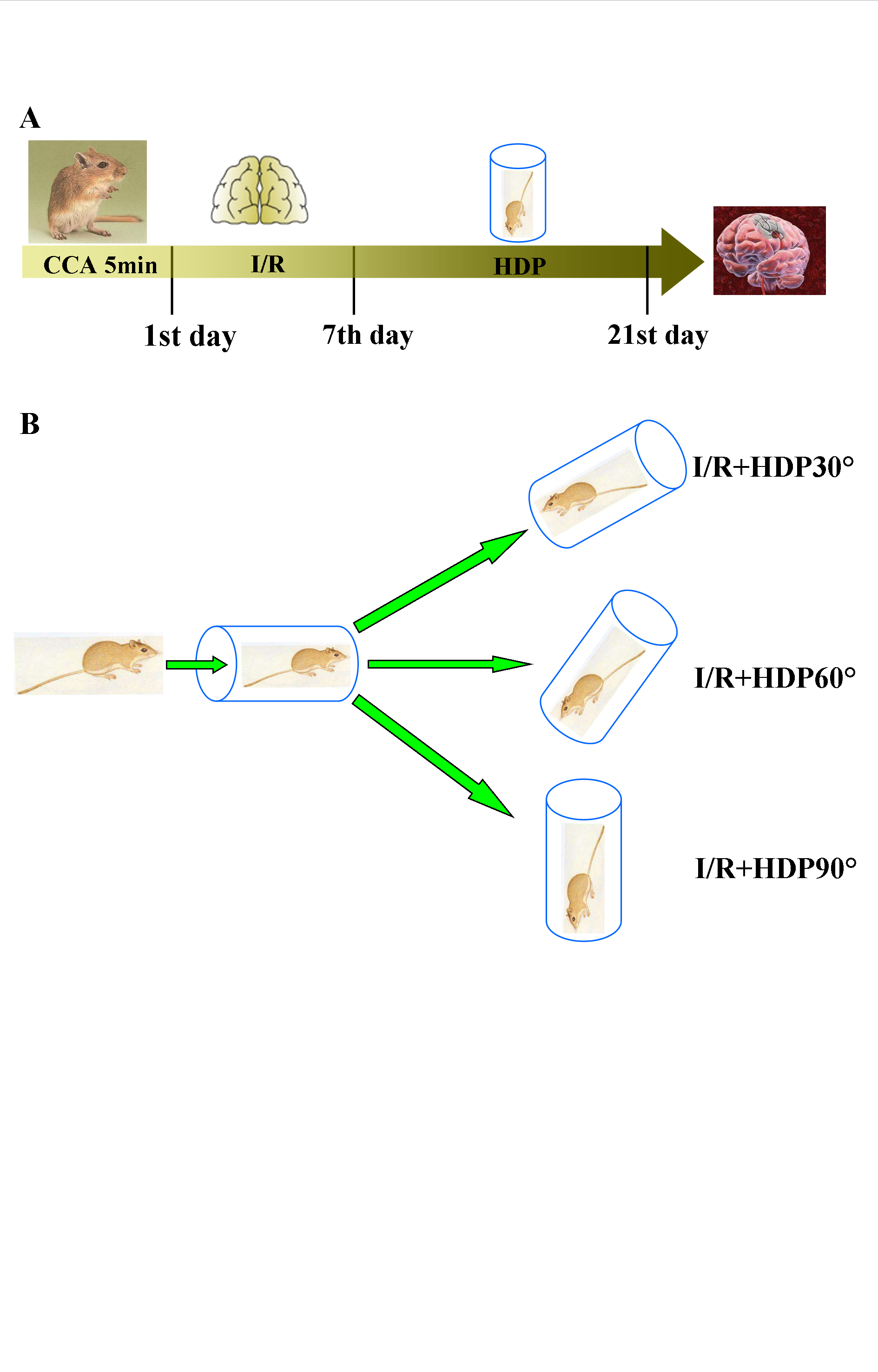

Supplement: Supplementary file 1 [file Image_1.TIF]

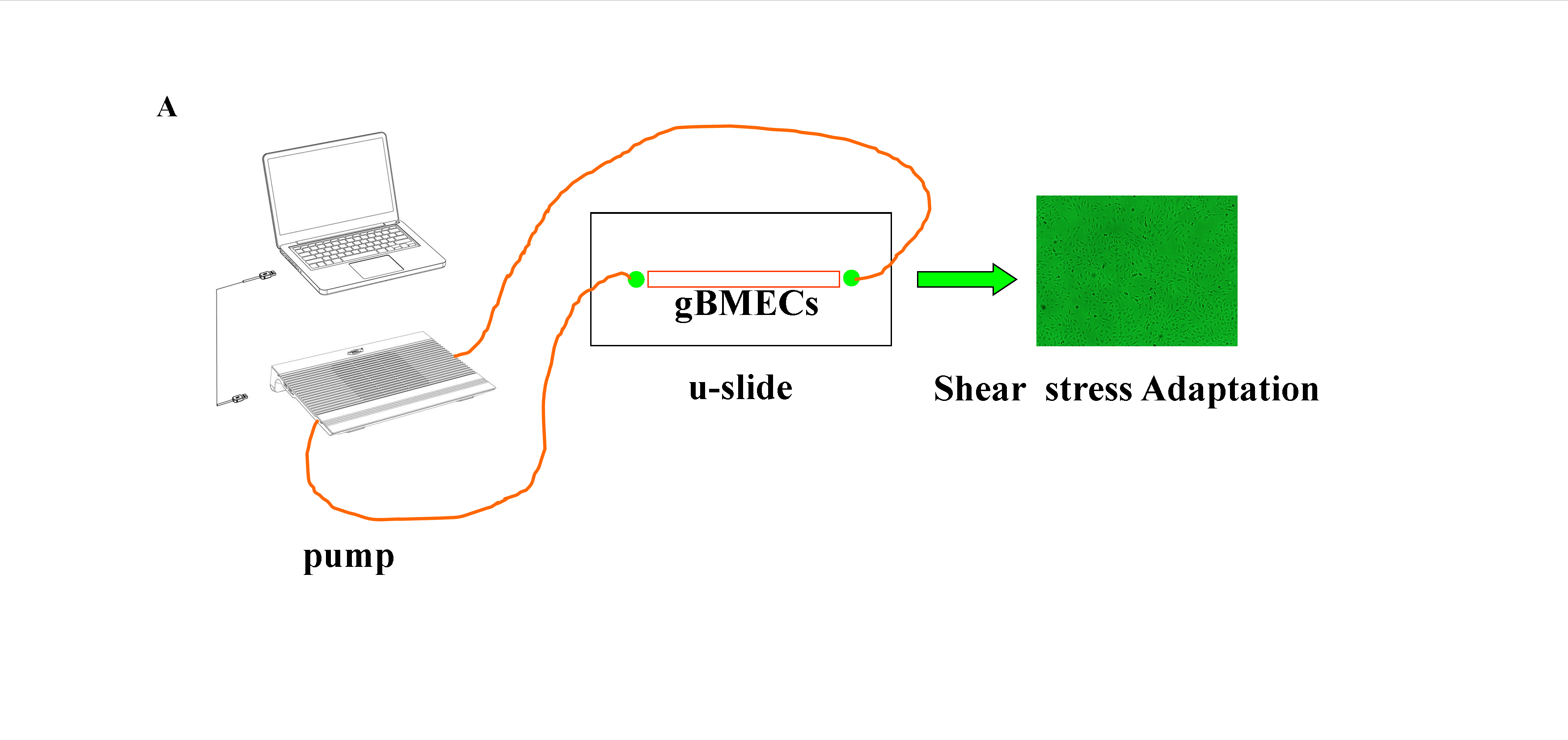

Supplement: Supplementary file 2 [file Image_2.TIF]
